# A scoping review of maternal health among resettled refugee women in the United States

**DOI:** 10.3389/fpubh.2023.1157098

**Published:** 2023-05-09

**Authors:** Sarah Yeo, Yuae Park, Deborah Jean McClelland, John Ehiri, Kacey Ernst, Priscilla Magrath, Halimatou Alaofè

**Affiliations:** ^1^Department of Health Promotion Sciences, Mel and Enid Zuckerman College of Public Health, University of Arizona, Tucson, AZ, United States; ^2^Department of Behavioral and Community Health Sciences, School of Public Health, University of Pittsburgh, Pittsburgh, PA, United States; ^3^Arizona Health Sciences Library, University of Arizona, Tucson, AZ, United States; ^4^Epidemiology and Biostatistics Department, Mel and Enid Zuckerman College of Public Health, University of Arizona, Tucson, AZ, United States

**Keywords:** refugee, prenatal care, maternal care, maternal health, United States, refugee health, host country, perinatal care

## Abstract

**Background:**

Globally, refugee women continue to face higher maternity-related risks from preventable complications during pregnancy and childbirth, partly due to high health care costs, unfamiliarity with the healthcare system, language barriers, and discrimination. Nevertheless, there is still a paucity of literature that evaluates the available evidence in the US. This scoping review delineated the body of literature on maternal health among refugee women resettled in the US in order to identify knowledge gaps in the literature and highlight future research priorities and directions for maternal health promotion.

**Methods:**

Electronic databases were searched in PubMed, CINAHL, PsycINFO, and EMBASE from inception through July 2021. We included all peer-reviewed study designs; qualitative, quantitative, and mixed method if they reported on refugee women's perinatal health experiences and outcomes in the US.

**Results:**

A total of 2,288 records were identified, with 29 articles meeting the inclusion criteria. Refugee women tend to initiate prenatal care late and have fewer prenatal care visits compared to women born in the US. Some of them were reluctant to get obstetric interventions such as labor induction and cesarean delivery. Despite numerous risk factors, refugee women had generally better maternal health outcomes. Studies have also highlighted the importance of health care providers' cultural competency and sensitivity, as well as the potential role of community health workers as a bridge between refugee women and health care providers.

**Conclusions:**

The scoping review emphasizes the need for early prenatal care initiation and more frequent prenatal care visits among refugee women. Furthermore, more needs to be done to mitigate resistance to obstetric interventions and mistrust. The mechanism by which healthy migrant effects occur could be better understood, allowing protective factors to be maintained throughout the resettlement and acculturation process. The scoping review identifies critical gaps in the literature, such as the underrepresentation of different ethnic groups of refugee women in refugee maternal studies in the US. Since this invisibility may indicate unspoken and unaddressed needs, more attention should be paid to underrepresented and understudied groups of refugee women in order to achieve health equity for all.

## 1. Introduction

Globally, persecution, conflict, violence, and human rights violations forced more than 26.6 million refugees to flee their home countries by the end of 2020 (1). Refugees tend to have higher health needs, limited access to health care, and poorer health outcomes. In addition, resettled refugees also experience challenges accessing health care in a host country due to high costs associated with care, limited health literacy, unfamiliarity with the new healthcare system, language barriers, inadequate health insurance, and racism and discrimination (2–6).

Refugee women, who account for nearly 47 percent of all those displaced across borders, are particularly vulnerable (1). Maternal health is adversely affected by a lack of housing, limited access to water and sanitation facilities, inadequate food and nutrition, and a lack of availability and accessibility of maternal healthcare services during forced displacement (7).

Migrant women, including refugee women, have higher maternal health risks than women in host countries, including gestational diabetes, stillbirth, low-birth-weight infants, early neonatal mortality, prenatal mortality, and preterm birth (8–10).

Refugee and migrant women have been found to attend fewer antenatal care appointments than their counterparts in host countries, despite the potential health risks (11). This is likely due to a variety of factors such as language barriers, cultural differences, transportation difficulties, financial constraints, and fear of discrimination (5, 11, 12). In the United States (US), research indicates that refugee and migrant women tend to postpone antenatal care visits more frequently than their domestic counterpart (11).

The US has welcomed refugees, resettling more than 3 million since the Refugee Act was passed in 1980 (13). Refugees admitted to the US are eligible for short-term health insurance known as Refugee Medical Assistance (RMA) for up to 8 months. Those who qualify for Medicaid may continue to receive benefits after the first 8 months (14). In addition, all refugees are entitled to a trained interpreter for medical visits. Health care providers receiving federal funds are mandated by Title VI of the 1964 Civil Rights Act to provide free interpretation services to those with limited English proficiency (15, 16).

Despite the resources available to admitted refugees, refugee health promotion programs in the US are often short-term, focusing on those who have been in the country for <2 years (17, 18). However, there is evidence that the refugees, the majority of whom had been in the country for more than 5 years, still had limited access to health care due to language barriers and a lack of insurance (19). Although the maternal health challenges experienced by refugee women resettled in the US are well-acknowledged, there remains a paucity of literature that critically summarizes available evidence. One study that synthesized systematic reviews conducted on perinatal health outcomes and care among refugees and asylum seekers identified 29 systematic reviews. Among these, no study focused solely on maternal health among refugees resettled in the US (9).

This scoping review was conducted to delineate the body of literature on maternal health among refugee populations resettled and living in the US. It sought to provide the magnitude, type, and nature of the studies through a thematic analysis, identify knowledge gaps in the literature, and highlight future research priorities for maternal health promotion among refugee women (20).

## 2. Methods

We included all peer-reviewed study designs, including qualitative, quantitative, and mixed methods. The definition of a refugee follows the one stipulated in the Immigration and Nationality Act (INA) “person who has experienced past persecution or has a well-founded fear of persecution on account of race, religion, nationality, membership in a particular social group, or political opinion” (13). The authors reviewed the papers based on the initial inclusion criteria, and as necessary, further details were added iteratively through consensus among the authors. More detailed inclusion criteria were as follows:

- Original research (excluding literature review, systematic review, scoping review, or policy guidelines)
- Studies that were conducted in the US
- Studies of maternal health care service utilization and maternal health outcomes among refugee women resettled in the US
- Topic is related to maternal care access and utilization among refugee women in the US

**The studies were excluded if they were:**

- Presentation abstracts without full texts
- Clinical reports
- Studies only related to gynecologic care, family planning, contraceptive care, or female genital mutilation
- Studies whose primary interests are infant or children without discussing maternal health, maternal health care access or utilization (studies that only examined child feeding or breastfeeding among refugee populations were excluded)
- Studies not directly related to pregnancy or maternal health although the population involves pregnant or postpartum refugee women (for example, HIV/AIDS, B hepatitis among pregnant refugee women)
- Studies without disaggregated data on refugee women.

The following electronic databases were searched with the assistance of a health science Librarian (DM): PubMed, CINAHL, PsycINFO, and EMBASE. The general concept areas searched were (1) refugee and (2) maternal. The search strategy was developed for each database including keywords and controlled vocabulary specific to the respective databases such as MeSH and Emtree. The databases were searched from inception through July 15, 2021. We did not apply any language or publication date restrictions to avoid potential bias associated with the restrictions (21, 22). The full search strategy is in Appendix.

After exporting the results from the searches, duplicates were removed using the function in EndNote. Then, the results were exported to Rayyan, a tool for systematic reviews (23). Two authors (SY and YP) independently screened the titles and abstracts of the records in Rayyan. The platform allows for blinded screening functionality so that one reviewer's decision would not influence the other reviewer. Throughout the review process, reviewers met on a regular basis to discuss the differences and reach a consensus. We intended to consult the other authors if any disagreements arose, but there were none.

The first author created a data extraction form to chart the results, which was validated through the screening process and modified as needed. After completing the form, two reviewers read the selected articles, entered the extracted data into the agreed form, and conducted a thematic analysis based on the form. The data were synthesized based on themes such as outcomes investigated and pregnancy period (prenatal, intrapartum, postpartum). Finally, we organized the studies and data by the identified themes, summarized the key findings, and discussed the implications.

We did not perform a quality assessment to exclude studies based on the results because the aim of this scoping review was to provide an overall picture of existing knowledge in relation to the topic of interest. The reporting followed the PRISMA guidelines.

## 3. Results

**Figure 1:**
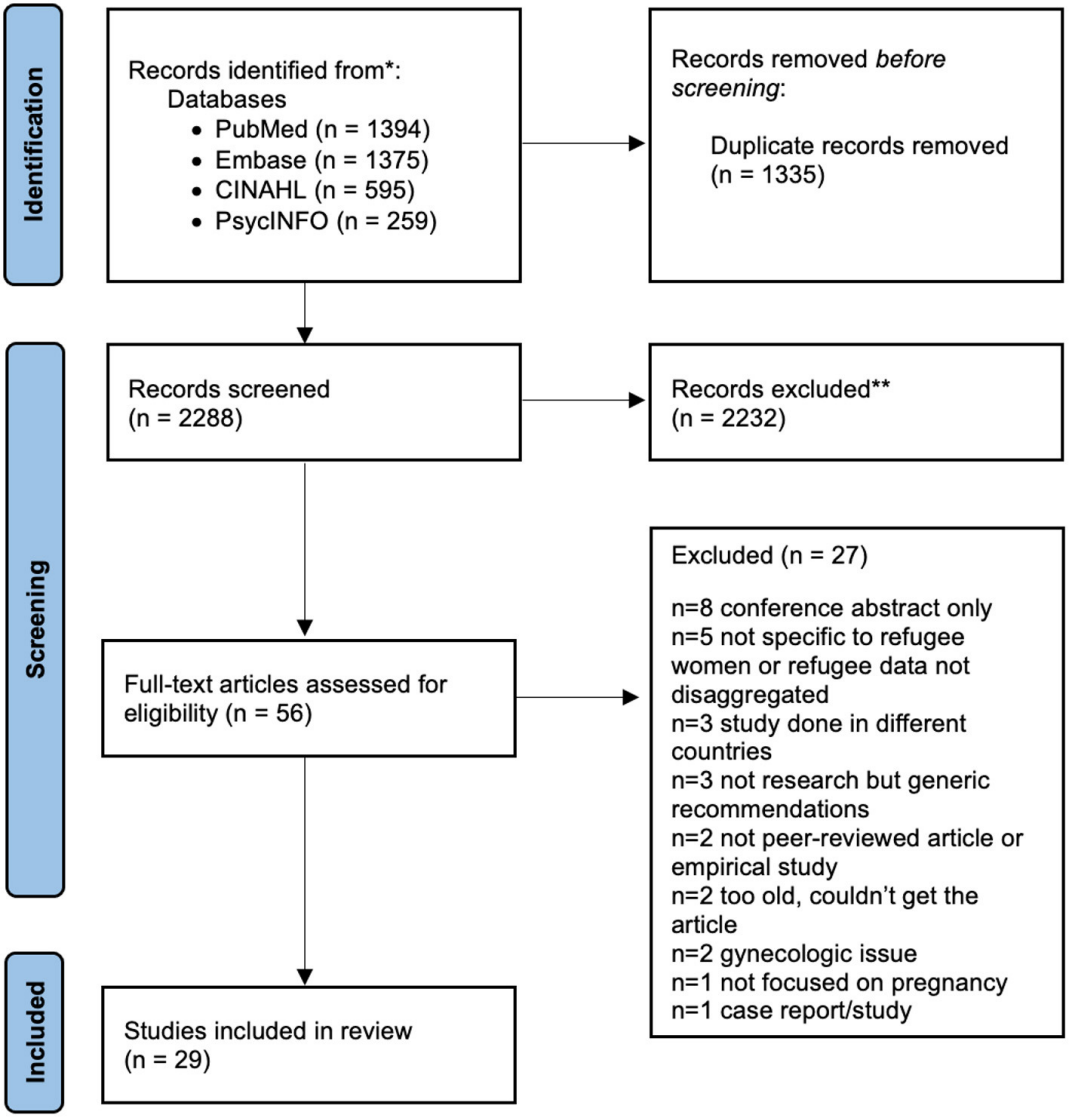
PRISMA flowchart of study selection.

A total of 2,288 records were identified, with 29 articles meeting the inclusion criteria (Figure 1). Seventeen of the articles included were quantitative, 11 were qualitative, and one was a mixed method study. One-third of the studies included were on Somalis, and one-fifth were on Indochinese/Southeast Asians. Most studies were conducted in Minnesota, followed by New York and Ohio (Table 1).

**Table 1:** Summary of the included papers.

**Characteristics**	**# (%)**
**Ethnicity**	
Somali	8 (28)
Indochinese/Southeast Asian	5 (17)
Mixed ethnic groups	5 (17)
Hmong	3 (10)
African	2 (7)
Bhutanese	2 (7)
Cambodian	1 (3)
Haitian	1 (3)
Karen	1 (3)
Tai Dam	1 (3)
**Geographical setting (States in the US)**	
MN	7 (24)
NY	5 (17)
OH	5 (17)
CA	2 (7)
MA	2 (7)
AZ	1 (3)
FL	1 (3)
GA	1 (3)
IA	1 (3)
MD	1 (3)
OR	1 (3)
WI	1 (3)
Mixed	1 (3)
**Study design**	
Quantitative	17 (59)
Qualitative	11 (38)
Mixed methods	1 (3)
**Years of publication**	
Prior to 2000	12 (41)
2000–2009	1 (3)
2010-present	16 (55)

To some extent, the studies on refugee maternal health appear to mirror the influx of refugees in the country caused by political turmoil and conflicts. As of 1981, in the aftermath of the Vietnam War and the fall of Saigon, ~500,000 refugees from the affected areas had fled to the US (24). Southeast Asians accounted for roughly one-third of annual deliveries in some of the US cities that received a high volume of refugees (25), and a small city with a total population of 97,000 at the time had ~16,000 Cambodian refugees (26). As a result, all studies published in the 1980's, with the exception of one, described maternal characteristics and outcomes, issues and cultural beliefs, values, and practices of Southeast Asians, and discussed challenges caused by new encounters with new immigrants and cultural and linguistic differences. The authors were mostly doctors and nurses who were grappling with the new challenges and most of the studies were conducted in the states that accepted a large number of refugees. The early 1990's were marked by the civil war in Somalia, and the US started resettling refugees from Somalia in 1990. Since then, Somali refugee arrivals have remained high with more than 100,000 refugees from Somalia during the last two decades (27). Out of all reviewed studies, eight studies, which is around 30%, were on Somali refugee women. Table 2 illustrates the overall characteristics of the included studies.

**Table 2:** Summary of included papers.

**Author**	**Year**	**Study aim**	**Design**	**Population**	**Site**	**Sample size**	**Conclusions**
Davis et al. (33)	1982	To look at the characteristics of the increasing numbers of births to the Indochinese	Quantitative	Southeast Asia	CA	Five hundred and forty two live births	The pregnancy outcomes of Indochinese were generally favorable, although the more recent arrivals, especially Cambodians and Laotians, present at greater risk, lacking prenatal care, and have more infants with low birth rates and more pregnancy complications.
Ellis (25)	1982	To describe issues and obstetric and nurse-midwifery service to Southeast Asian refugee families in the area	Qualitative	Southeast Asia	CA	Not available (but it seems to be based on the interviews with nurses and midwives)	The Southeast Asian refugee population has many challenges with late prenatal care, language barriers, issues with the insurance, cultural beliefs. However, it can be mitigated by mutual understanding and efforts.
Dempsey et al. (50)	1983	To assess the beliefs, values, and practices related to childbearing of the Haitian refugee in Miami	Qualitative	Haitian	FL	Ten refugee women	In general, the Haitian refugee women had similar concerns and perceptions of pregnancy to their American counterparts. The differences in their answers highlighted the necessity of assessment and care tailored to each woman.
Hopkins et al. (34)	1983	To evaluate the relationship between pregnancy risk factors and length of time the refugees were in the US prior to delivery	Quantitative	Southeast Asia	OR	Nine hundred and twenty one live births	Particularly for Hmong, higher maternal and newborn risks were observed. For refugees who have lived in the US for 3 months or more, data show increases in accessing prenatal treatment and a decrease in the number of low-birth-weight newborns; however, the advantages appear to be undone after 12 months of US residency.
Nelson et al. (24)	1983	To better understand their cultural practices, medical and obstetrical problems, and challenges	Quantitative	Southeast Asia	MN	Two hundred and twenty nine refugee women	Despite challenges such as language and cultural barriers, the obstetric and newborn outcomes for this group of women were good.
Swenson et al. (42)	1986	To compare maternal characteristics and pregnancy outcomes of Hmong and other Southeast Asian refugees, Caucasians and Black mothers	Quantitative	Southeast Asia	MN	Three hundred and thirty seven adolescents and 876 mothers including White and Black residents	In spite of the significantly late initiation of prenatal care, lower weight gains, and high prevalence of anemia, the virtual absence of alcohol and tobacco consumption among the population may contribute to their generally favorable pregnancy outcomes.
Edwards et al. (41)	1987	To compare pregnancy course and maternal and fetal outcome in this Hmong population with White, Black, Hispanic, and American Indian	Quantitative	Hmong	MN	Six hundred and eighteen Hmong refugee women (5,278 controls)	Given the risk factors, including indigence, advanced maternal age, grand multiparity, short stature, delayed prenatal care, poor nutrition, and anemia, the pregnancy and birth outcomes were largely favorable.
Erickson et al. (35)	1987	To compare selected maternal characteristics and infant outcomes of the Hmong, a subgroup of Southeast Asian refugees, with Caucasians	Quantitative	Hmong	MN	Three hundred and fifty Hmong and 573 Caucasian mothers	Despite having higher parities, delayed prenatal care initiation, and lower hematocrits at 36-week exams, Hmong mothers did not differ significantly from their Caucasian counterparts regarding gestational age, Apgar scores, or low birthweight babies.
Bell et al. (45)	1987	To discuss several aspects of health care utilization and health care practices for a group of Tai Dam women	Quantitative	Tai Dam from Southeast Asia	IA	Fifty two refugee women	A number of issues were noticed, including Tai Dam cultural views about illness, inadequate health insurance, and communication problems.
Gann et al. (26)	1989	To define the prevalence of adverse birth outcomes and to describe the major risk factors in the Cambodian refugee women	Quantitative	Cambodian	MA	Four hundred and fifty two consecutive pregnancies among Cambodian women and 110 low-income White individuals	Despite the prevalence of multiple risk factors, major pregnancy problems were less frequent in this population, and low birthweight prevalence was close to the state average.
Schauberger et al. (28)	1990	To review aspects of obstetric care and outcomes specific for the Hmong refugees	Quantitative	Hmong	WI	Four hundred and thirty five babies delivered	The data revealed a population with a wide range of medical issues but generally positive obstetric outcome.
Kahler et al. (44)	1996	To evaluate the access, timing, and frequency of prenatal care and clinical findings in the refugee populations	Quantitative	Mixed	NY	Fifty nine pregnant women	Refugees from low-income nations may have ailments that need to be diagnosed and treated to ensure a healthy pregnancy. Due of the possibility of poor pregnancy outcomes, pregnant women were a major source of concern.
Herrel et al. (36)	2004	To understand experiences of Somali women concerning pregnancy and childbirth and determine the childbirth education needs	Qualitative	Somalia	MN	Forteen refugee women	Despite reporting a positive childbirth experience, the participating women mentioned racial stereotyping, concern about C-sections, and the competence of medical interpreters. They also expressed the need for more information and strategies to encourage them to attend prenatal checkups.
Brown et al. (38)	2010	To examine sources of potential dissatisfaction and resistance to prenatal and obstetrical interventions among resettled Bantu Somali and non-Bantu Somali women	Qualitative	Somalia	NY	Thirty four refugee women	There was significant opposition to a variety of obstetrical procedures, including C-sections. To improve maternity care for Somali women, it is essential to educate both Somali women and medical personnel.
DeStephano et al. (29)	2010	To determine the level of acceptability of health education videos by Somali refugee women in a clinical setting and health providers' perceptions regarding usefulness of the videos	Mixed methods	Somalia	MN	Twenty two refugee women	Women preferred video health education materials delivered in the Somali language, and video formats for prenatal education seemed suitable to Somali patients.
Flynn et al. (49)	2011	To explore whether acculturation has affected Somali women's birth outcomes over time	Quantitative	Somalia	MN	Five hundred and eighty four refugee women	Over time, there were notable increases in substance use and exposure, interpreter use, body mass index, hemoglobin levels, gestational diabetes, and preterm birth. Although acculturation-related traits are becoming more prevalent among Somali women, they do not entirely account for the rise in preterm birth.
Lazar et al. (39)	2013	To explore providers' experiences, practices, and attitudes toward prenatal care and delivery of women with female genital cutting (FGC)	Qualitative	Somalia	OH	Fourteen health care providers	Establishing effective communication, enhancing providers' clinical and cultural training, enhancing health literacy, and building trust through community engagement are all necessary for improving the clinical encounter for both patients and providers and reducing frustration, disappointment, and mistrust.
Johnson-Agbakwu et al. (37)	2014	To examine the perspectives of Somali men toward FGC and women's childbirth experiences in one refugee community in the USA	Qualitative	Somalia	AZ	In phase 1, three focus groups with a total of 32 men. In Phase 2, eight men who had participated in the initial focus groups were purposively selected	The perspectives of Somali men regarding their experiences giving birth include strong matriarchal support for FGC, discomfort with men present during labor, and a strong reluctance to cesarean delivery.
Miller et al. (32)	2016	To examine whether immigrants and mothers from refugee countries have lower adjusted risk of preterm births than US-born mothers in Syracuse, NY	Quantitative	Mixed	NY	Six thousand and three hundred and fifty four birth records (966 infants born to foreign-born mothers, 575 of whom were from countries of origin for refugees living in Syracuse and 5388 infants born to US- born mothers)	After adjusting for confounders, babies born to foreign-born mothers and to women from refugee countries were less likely to be born prematurely than babies born to US mothers.
LaMancuso et al. (53)	2016	To study the perspectives of Karen refugee women in Buffalo, NY, their medical providers, and Karen interpreters/doulas on perinatal care for Karen women in resettlement	Qualitative	Karen	NY	Twenty eight interviews: 14 Karen patients, 8 Karen doulas and community leaders, and 6 representatives from the clinic	Karen women's seeming agreeability may be attributed to low self-efficacy, traumatic experiences, and cultural expectations. Doulas/interpreters help patients and the care team communicate by having insider knowledge of women's issues.
Kentoffio et al. (30)	2016	To determine use of recommended maternal healthcare services among refugee and immigrant women in a setting of near-universal insurance coverage	Quantitative	Mixed	MA	Three hundred and seventy five women with 763 pregnancies (women/ pregnancies: 53/116 refugee, 186/368 Spanish-speaking immigrant, 136/279 US-born control)	Refugee and immigrant women had increased risk for delayed initiation of prenatal care, but greater use of postpartum visits.
Van Zandt et al. (52)	2016	To provide a description of the outcomes for vulnerable groups of mothers and newborns served by the Birth Companions Program	Quantitative	Mixed	MD	One hundred and forty four refugee women (a total of 1,511 including comparisons)	There were no differences between the groups regarding cesarean deliveries, Pitocin induction/augmentation, low birth weight, or premature babies. This result may be affected by interventions by the Birth Companion.
Kingsbury et al. (47)	2018	To describe the social networks of Bhutanese refugee women who gave birth in the US to better understand how supportive these relationships were for refugee women during their pregnancies	Qualitative	Bhutanese	OH	Forty five refugee women who had given birth in the past 2 years	Most of the close relationships formed by participants during their pregnancies were with female family members they knew before moving to the US. In addition, participants considered their social networks very close and felt supported by them throughout their pregnancies.
Agbemenu et al. (43)	2019	To compare prepregnancy health and prenatal behavior and prenatal history and prenatal care utilization and labor and birth outcomes between African refugee women and US-born Black and White women	Quantitative	Africa	NY	Seven hundred and eighty nine refugee women (as compared to 59,615 US-born white women and 17,487 US-born Black women)	African refugees experienced more favorable health outcomes than US-born groups despite later initiation of prenatal care and lower scores of prenatal care adequacy.
Kingsbury et al. (48)	2019	To examine the presence of social support among Bhutanese refugee women during their pregnancies and to determine which demographic characteristics are associated with strong social support	Quantitative	Bhutanese	OH	Forty five refugee women	Personal social networks are an important source of support for resettled refugee women during pregnancy in the US. Refugee women who experience secondary resettlement may perceive stronger support from their personal connections.
Banke-Thomas et al. (31)	2019	To examine maternal and reproductive health access of Somali refugees in the US across four access dimensions (willingness to seek care, gaining entry to the health system, seeing a primary provider and seeing a specialist)	Quantitative	Somalia	OH	Four hundred and twenty seven refugee women	Lack of insurance, limited language fluency and being circumcised limited access to care across all dimensions.
Khan et al. (46)	2019	To identify effective delivery methods of prenatal, postnatal, and family planning services to refugee women, and the challenges or barriers to providing these services from an organizational perspective to inform policy on refugee maternal health	Qualitative	Mixed	Mixed	Five staff from five refugee serving organizations (including a director, nurse case manager, midwife team leader, and CEO)	Focusing on client capacity building, individualized support, successful collaborations, and cultural sensitivity was key to success. Respondents noted the need for physical resources, strong leadership, and more staff, particularly with linguistic skills. The necessity of funding and laws supporting respondents' work was emphasized.
Fuller et al. (51)	2021	To evaluate a video-based curriculum to improve refugee women's birth experiences in the US	Qualitative	Africa	GA	Not available	The videos using native speakers with experiences similar to current pregnant refugee participants were a powerful intervention for supporting birth preparedness.
Agbemenu et al. (40)	2021	To identify perceived protective mechanisms used to avoid obstetric interventions as well as the underpinning factors that influence aversion to obstetrical interventions by Somali refugee women	Qualitative	Somalia	OH	Forty refugee women	Somali women's mechanisms to avoid obstetrical interventions necessitate building trust, addressing the fears, and respecting their cultures.

The following sections present a summary and critical appraisal of key findings of the included studies.

### 3.1. Maternal health care utilization

#### 3.1.1. Prenatal care

Overall, refugee women tend to initiate prenatal care late and have fewer prenatal care visits than women born in the US (28–32). In the early 1980's, studies on Indochinese women consistently found that refugee women delayed seeking prenatal care. Many women delayed seeking prenatal care until the second or third trimester or did not seek prenatal care at all (25, 33, 34). One study on the Hmong refugee women noted a similar pattern with 16% refugee women beginning care in the first trimester, compared with 44% of the White women (35). A more recent study also indicated that 20.6% of refugee women had their first prenatal care visit after the first trimester, compared with 6% of women born in the US (30). The authors suggested that although women may recognize the importance of prenatal care visits, (36). refugee women may not be fond of visiting hospitals or perceive that a hospital is only for the sick (37). Or they may be reluctant to reveal their pregnancy and seek advice from elders rather than health care providers (37). According to one study, Hmong refugee women are reluctant to undergo procedures including pelvic exams, blood draws for testing, and the use of iron supplements since these practices violate their cultural traditions (35). These perceptions, preferences, and cultural norms all appeared to contribute to refugee women's delayed prenatal care visits.

#### 3.1.2. Intrapartum

One theme that stood out in the scoping review was avoidance and resistance toward intrapartum interventions particularly among Somali women (36–40). and Southeast Asian women. The opposition seemed deeply rooted in the perception that health care providers prefer the interventions, such as induction and cesarean delivery, as “they were quicker, more convenient, or more lucrative” (39). Or Somali women considered the intervention to be the result of health care providers' ignorance of how to properly care for women with female genital cutting (FGC) (37). There were also studies on Southeast Asian refugee women that demonstrated their resistance to “unnatural” practices (24, 28, 35). They frequently held the view that cesarean birth and episiotomies were unnatural procedures with unfavorable outcomes. Some people believed that having a C-section is an indication of their female incapacity and a lasting physical disability (24).

#### 3.1.3. Postpartum

A recent study comparing the use of prenatal and postpartum health services among refugee women and women born in the US found that refugees were more likely to have postpartum visits (73.3% and 54.8%, respectively) although the study does not elaborate on the reason (30).

### 3.2. Maternal health outcomes

#### 3.2.1. Prenatal

Two studies reported that hypertension and preeclampsia were less likely to be observed among refugee women (28, 41). However, one study of Hmong women discovered that they were five times more likely to have experienced previous perinatal loss than women in the host country (41). Also, the other study speculated on the possibility of spontaneous miscarriages at home without seeking medical attention (28).

#### 3.2.2. Intrapartum

Studies on Southeast Asian refugee women noted that the prevalence of cesarean delivery was significantly lower in refugee women compared to US-born mothers (24, 26, 28, 33, 41, 42). Also, fewer obstetric interventions such as induced labors and fetal monitoring were observed among Indochinese refugee women (42). African refugee women were less likely to experience cesarean delivery and induced delivery than US-born white and black mothers. The vaginal birth was 73.4% among refugee women as compared to 65.3% US-born white and 66.6% US-born black mothers) (43). Induced delivery occurred in 19.1% of refugees, 29.7% of US-born white mothers, and 25.6% of US-born black mothers (*p* < 0.001) (43). Another study, which looked at multiple ethnic groups of refugees, found the opposite. In this study, the aggregated refugee group had a higher prevalence of cesarean section (24.3%) than the US-born control (17.9%) or the other immigrant group (17.4%), though the difference was not statistically significant in the adjusted model (30).

#### 3.2.3. Postpartum

Refugee women had generally better maternal health outcomes than US-born women despite risk factors such as delayed initiation of prenatal care, lower weight gains, poor nutritional status, psychosocial factors, high prevalence of infectious diseases, anemia, and short stature (26, 32, 33, 35, 41–43). One study found that Cambodian refugees had a lower prevalence of low birthweight infants, stillbirths, or major pregnancy complications than White low-income women in the state (26). A similar trend was observed in the study of Hmong refugee women. Despite risk factors such as high parity and delayed prenatal care, few low birthweight infants were born to Hmong mothers (35). Prematurity and perinatal mortality rates were also low (41). Another study on Indochinese refugee mothers noted that their low birthweight rates were slightly lower than the comparison (5.7% and 7.1%) and that their median birthweight (3,175 gm) is equivalent to that of mothers in the US. They also had a reduced infant mortality rate (33). A more recent study with mixed ethnic groups reported that refugees had a lower risk of preterm birth than US-born mothers, with an adjusted relative risk of 0.67 (95% CI 0.49–0.89) (32). Another study on African refugee women by Agbemenu et al. also found that African refugee women had fewer preterm births (*p* < 0.001), fewer low birth weight infants (*p* < 0.001), and higher rates of vaginal deliveries (*p* < 0.001) compared to their US counterparts (43). In one study of Indochinese women, the mean birth weight of the refugee women was 350 gm less than the mean for infants born to US-born mothers, but the mean was 3,200 gm, which was within the normal range (24). One study, however, concluded that prematurity was more common among the infants born to the Hmong refugees (8%) than the comparison (4%) (*p* < 0.01) (28).

### 3.3. Risk factors

The reviewed studies included different levels and domains of risk factors; biological factors such as maternal age, stature, nutritional levels, anemia, preexisting medical conditions, pre-pregnancy body mass index, behavioral risk factors in pregnancy such as smoking, drinking, and drug use, psychological factors, cultural factors, and social determinants of health such as insurance and socioeconomic status. Below is a summary of key themes identified from this review.

#### 3.3.1. High prevalence of infectious disease

Infectious diseases were prevalent among refugee women who had resettled early in the 1980's (24, 25, 33). In a study with mixed ethnic groups, various infectious diseases were common among the women, including urinary tract infections (26% among African women and 14% among Central American women), monilial vaginitis (29% among African women and 29% among Central American women), tuberculosis (51% among African women, 41% among Central American women, 40% among Sri Lankan women) (44). Multiple parasitic infestation and hepatitis were also prevalent (24, 25, 33). However, a 1990 study of Hmong refugee women found that parasitic infections and hepatitis were less common than previously, indicating that many resettled refugee women in 1979–80 came from refugee camps with poor living conditions (28).

#### 3.3.2. Anemia

Anemia was also common among refugee women, which could be attributed to chronic malnutrition, untreated hookworm infestation, or malaria (24, 26, 28, 42, 44). In a study conducted by Kahler et al. (44), a majority of refugee pregnant women had anemia ranging from 67% (women from Central America) to 88% (women from Africa).

#### 3.3.3. Access to insurance

In two studies, almost one quarter of studied refugee women were not covered by any medical insurance (31, 45). In one study that examined reasons for postponing care among Somali refugees, 81% indicated that not having insurance coverage was a reason for postponing care (31). Also, it was noted that unapproved specialist care was a barrier to care in the same study. Those with health insurance, whether public or private, were more likely to seek care, enter the health system, experience less difficulty in meeting a primary care provider and a specialist compared to those without (31). Another study underscored the importance of having medical insurance for return visits after the initial visit (45).

#### 3.3.4. Female genital circumcision

Compared to women without FGC, Somali refugee women with FGC were 50% less likely to seek medical care. Another factor was severity, with more severe FGC types being linked to lower motivation to seek care and higher access and entrance barriers to care (31, 39). The needs for training to provide “non-judgmental” and optimal care for women with FGC were noted. In **one** study that interviewed 14 health care providers who serve Somali refugee women, only **one** provider responded that she had received any type of formal training on the management of women with FGC prenatally and during labor and delivery (39).

#### 3.3.5. Maternal age

Refugee women tend to have advanced maternal age compared to US-born women (26, 41). In a study conducted by Edwards et al., Hmong mothers were seven times more likely to have geriatric pregnancy than the comparison group (41).

#### 3.3.6. Social determinants of health

According to a study, mothers of refugees had less years of education than mothers who were born in the US (11 years of education for the white and black mothers on average and 2 years for the Hmong and 5 years for other Southeast Asians) (42). Another study based on secondary analysis of birth records of mothers from “refugee countries” indicated that they were also more likely to have low socioeconomic status (32). One study on Tai Dam refugee women noted that they were likely to work as full-time housewives or work in a blue-collar job if employed (45). According to one recent study on Somali women, more than half of the surveyed women lived in households that were below the poverty line (31).

#### 3.3.7. Dietary patterns during pregnancy

Southeast Asian refugee women reported two dietary patterns during pregnancy: restricting food intake and reluctance to take medications. Many Southeast women ate only once or twice a day because they were afraid of gaining too much weight and having a difficult delivery (25, 35). Additionally, some pregnant women were hesitant to take drugs like iron and vitamin supplements for concern that the baby could become too big (24, 35).

#### 3.3.8. Language barriers

Many studies frequently mentioned language as a barrier to quality care. Women often had limited English proficiency regardless of length of stay in the US, and many women particularly from Southeast Asia in 1980's were illiterate in their native languages (24, 25, 31, 33, 41, 42, 44, 45). Even when interpretation services were available, women would not freely communicate with male interpreters, particularly during prenatal exams, labor, or delivery (24). Unfamiliar disease names and medical terminology added complexities (24). The quality and objectivity of the interpreters were also questioned at times (39).

#### 3.3.9. Lack of medical records and documentation

Medical history including past pregnancy losses were not properly documented and other important information such as length of gestation, menstrual history, and the data of the last menstrual period was often not known (24).

#### 3.3.10. Mistrust toward health care providers

Patients' mistrust of healthcare professionals was noted, especially among Somali patients (37, 39). The significant mistrust resulting from conflicting cultural norms and views seemed to stand in the way of effective patient-provider dialogue and high-quality care (37, 39).

#### 3.3.11. Decision making

Another theme that came out in terms of maternal health among refugee women was decision making process. Health care decisions concerning refugee women's health were frequently made by the husband or head of the family, potentially due to patriarchal social norms, leading to frustration among providers who want direct communication with the women and her own autonomy (24, 39).

#### 3.3.12. Political uncertainty

While the majority of the studies we analyzed discussed risk factors on a micro level, one research focused on political uncertainty, a risk factor on a macro level. According to Khan et al. (46) one of the difficulties in maintaining program stability was political unpredictability when they spoke with service providers who assist refugee women. Service providers commonly believed that the uncertainty posed a danger to the funding and sustainability of their programs because the number of refugees relocated is decided by a Presidential Executive Order each year and is frequently impacted by political climates.

#### 3.3.13. Discrimination

One of the barriers was racial discrimination by healthcare professionals, according to research by Herrel et al. (36) on Somali refugee women.

### 3.4. Protective factors/enablers

#### 3.4.1. Social support and network

As social support may influence maternal health, one study investigated the social networks of Bhutanese refugee women who had given birth in the US (47). The women's most important networks during pregnancy were their spouses, female family members, and friends (71.9%). The authors concluded that these “low-heterogeneity” connections are frequently important sources of information, advice, and support during the resettlement experience (47). Another study published the following year by the same authors reached a similar conclusion. Notably, women who relocated from one location in the US to another were almost five times more likely to report having a strong support network, with an odds ratio of 4.52 (95%CI 1.19–17.15) (48).

#### 3.4.2. Low prevalence of smoking and drinking

Many studies found a low prevalence of smoking or drinking among refugee populations, pointing to a possible link between this and favorable maternal health outcomes despite adverse risk factors (26, 32, 35, 41, 42). In a study of Hmong refugee women in 1987, Hmong mothers did not use alcohol or smoke prior to or during their pregnancies, whereas 33.9% of Caucasian women used alcohol and 63.9% smoked (35). A similar trend was observed in a more recent study, though the figures were lower. When compared to refugee women, US-born mothers were also more likely to smoke during the first trimester (28.5% vs. 1.0%), use drugs (10.5% vs. 0.2%), and drink alcohol (1.4% vs. 0.3%) (32).

#### 3.4.3. Acculturation

In one study, Somali refugee mothers were examined to see how acculturation affected birth outcomes including gestational age and infant birthweight. Acculturation was measured using factors such as the age of immigration, years spent in the US, percentage of life spent there, and use of an interpreter at prenatal checkups, and the study concluded that it was not linked to preterm birth or low birthweight of infants in the study (49).

### 3.5. Cultural issues concerning maternal health and services

#### 3.5.1. Cultural norms and practices concerning pain

Culturally acceptable norms and practices during the intrapartum period may cause difficulties. For example, one study on Indochinese refugee women demonstrated how cultural norms relating to crying out in pain as shameful and pain tolerance as a virtue can make it difficult for health care providers to determine the delivery progression (25). This tendency was also observed in a study on Hmong women, which noted that because they are less likely to express pain and feelings during labor, special attention should be paid to subtle changes to assess progress (24).

#### 3.5.2. Preference for female health care providers

Refugee women preferred female health care providers across cultures and regions (24, 42, 50).

#### 3.5.3. Cultural practices and beliefs in relation to foods after delivery

Studies on Indochinese refugee women and Haitian refugee women noted cultural practices concerning foods for pregnant and postpartum women. For Indochinese postpartum women, only hot foods were allowed such as spicy tea or soup made with ginger root while prohibiting cold foods (25). The Haitian women also had to drink hot beverages such as hot milk or hot ginger tea during labor (50).

#### 3.5.4. Women kept warm after delivery

Cultural practices to keep postpartum women warm were noted in studies on Indochinese refugee women and Haitian refugee women (25, 50). After birth, the Vietnamese, Mien, and Cambodian were traditionally placed near a fire. Postpartum Cambodian women would cover their heads with a towel. To meet the needs of the postpartum women, the midwives in the study provided them with a gown and blanket directly from the warmer (25). The results of the other study showed that Haitian moms made an effort to avoid cold foods and beverages after giving birth (50).

## 4. Recommendations

Recommendations suggested from the reviewed studies are epitomized in the following.

### 4.1. Cultural competency and sensitivity of health care providers

Several studies emphasized the importance of building cultural competency and sensitivity of health care providers. The authors argued that health care providers need to be better informed and taught on cultures, traditions, values, expectations and reluctance of refugee women to certain medical interventions such as cesarean delivery (36). Moreover, communication based on the cultural competency will help mitigate deep-rooted distrust between providers and patients and contribute to quality care for refugee women (38, 39).

### 4.2. Culturally appropriate health education program and materials

The needs for culturally appropriate health education programs and materials were also noted.

Community-based education programs that are collaboratively developed and incorporate both US practices and refugee women's perspectives and cultures could mitigate women's apprehension regarding obstetrical interventions and contribute to better maternal health outcomes (38). Also, given the high illiteracy or limited English proficiency among refugee women, culturally tailored visual materials in their languages, such as education video series, were suggested to facilitate better communication between providers and patients (29, 36, 51).

### 4.3. Community health educators as a bridge between refugee women and providers

Several studies have emphasized the importance of community health educators, community health workers, or “cultural health navigators” as a potential bridge between the US health care system and refugee women. The studies, whatever the workers were called, revealed similar expectations for their roles. They could disseminate information and alleviate women's fears concerning certain obstetrical interventions (38, 52). They could facilitate communication between providers and patients (37). They could coordinate, providing reminders for the appointments and helping women comply with the care (30). According to one study, trained doulas of the same ethnicity as refugee women could also serve as a role of interpreters and advocates who “translate the cultures,” bridging the communication gap (53).

### 4.4. Better communication between health care providers and patients

Given their unfavorable attitudes of obstetrical procedures, one study that included the viewpoints of healthcare professionals that serve Somali people highlighted the importance of increasing communication between professionals, patients, and their families during antenatal care (39). The authors also suggested soliciting feedback from the Somali community and engaging them in the decision-making process in order to improve community health literacy and demystify fears (39).

### 4.5. Interventions to promote prenatal care visits

Several interventions were proposed to increase prenatal care visits, including prenatal appointment reminder calls, transportation to the appointment, and childcare assistance (36).

### 4.6. Empowering refugee women

The importance of empowering women was mentioned so that they could understand their rights to health care, leading to better decision making and health outcomes (46).

### 4.7. The need for funding, personnel, and increased interpretation resources

In a study which involved service providers for refugee women, most respondents mentioned the need for more funding, personnel, and interpretation resources to improve service quality (46).

## 5. Discussion

This scoping review delineated the existing evidence base of perinatal health outcomes and maternal health care experiences among refugee populations in the US. Overall, refugee women had a variety of risk factors, including a high prevalence of infectious diseases, anemia, a lack of insurance, and language barriers, and they were likely to delay prenatal care initiation and had fewer prenatal care visits compared to women in the US. They were opposed to obstetric interventions such as labor induction and cesarean delivery. Despite risk factors and inadequate prenatal care, however, refugee women had better maternal health outcomes than US-born women. A number of studies have associated it to the low prevalence of smoking and drinking. Nonetheless, refugee maternal health necessitates a more nuanced understanding due to heterogeneity and underrepresentation, a lack of aggregation, and a lack of data at multiple levels and trajectories of health outcomes among refugee populations, as described below.

### 5.1. Geographically and ethnically disproportionate focus on the issue

Although the studies reflect the majority of the trends in terms of incoming refugees in the US, the results highlight an evidence gap in terms of refugee maternal health, as well as a geographically and ethnically disproportionate focus on the issue. Table 3 depicts the number of refugees resettled in the country over the last two decades. Even though a large number of refugees from Burma, Iraq, the Democratic Republic of the Congo, or Iran have resettled in the US during the period, little is known concerning their access to maternal health services or maternal health outcomes. There is also a scarcity of literature on the maternal health status of the approximately 500,000 refugees resettled from former Soviet Union countries between 1983 and 2004 after the collapse of the Soviet Union (54).

**Table 3:** The number of refugees resettled in the US (FY2001-FY2022) (27).

**Country of origin**	**Number of refugees**
Burma	182,154
Iraq	148,167
Somalia	109,625
Bhutan	96,199
Dem. Rep. Congo	82,997
Iran	55,943
Ukraine	50,412
Cuba	49,575
Sudan	30,228
Russia	27,127
Syria	26,540
Liberia	24,417
Eritrea	22,444
Afghanistan	21,803

In terms of refugee maternal studies, some states were also underrepresented. According to recent data, five states received one-third of all refugees resettled in the US over the last decade: Texas (10%), California (9%), New York (6%), Michigan (5%), and Arizona (4%) (55). Despite the large number of refugees resettled in the states, no studies have been conducted on the maternal health of refugee women in Texas or Michigan, and the access to, utilization, or health outcomes of maternal health services in the states are largely unknown.

### 5.2. The need to recognize heterogeneity within a seemingly homogeneous group

Several studies focused on heterogeneity within a seemingly homogeneous group. Despite being grouped as “African refugees,” disparities were observed between groups of refugees from different African countries. In terms of highest level of education, English proficiency, and reported employment, resettling Congolese women and Somali women differed substantially (56). Indochinese refugees had varying levels of education, exposure to formal medical care and Western culture and different levels of maternal care utilization (24, 34). Furthermore, the Bantu Somali, although often lumped in with the rest of the Somali refugees, have distinct cultural, linguistic, and ethnic differences that set them apart from the rest of the Somali refugees (38). Different cultures, norms, pre-migratory experiences, and socioeconomic circumstances may result in varying levels of integration and acculturation, as well as access and utilization of maternal care services, satisfaction and trust in the health care system and health care providers. As a result, a greater emphasis should be placed on the subtle differences between seemingly homogeneous groups, such as refugee women or Somali refugees, and the various pathways through which each of them navigates health care systems and interacts with the system.

### 5.3. Disaggregated reporting

As previously stated, there is a great deal of heterogeneity among refugee women in general, and within a seemingly homogeneous group such as Somalia or Indochinese depending on ethnic groups. Different socioeconomic statuses, pre-migration experiences, pre-existing health conditions, living conditions prior to migration shape refugee women's perceptions and experiences in the US, and potentially lead to different maternal health outcomes. Furthermore, researchers must consider the possibility of a power dynamic among refugee communities, as well as pre-existing differences and disparities in access to resources and opportunities, all of which may influence health outcomes and create disparities. As a result, where possible, it may be useful to further disaggregate the ethnic groups, even within the commonly used category such as African refugees or Somali refugees.

### 5.4. Healthy migrant effects yet to be better understood

Despite the risk factors that refugee women in the US frequently face, favorable maternal health outcomes have been observed in many studies involving various ethnic groups in the scoping review (32, 41–43, 49). As previously stated, African refugees had fewer preterm births, fewer low birthweight infants, and higher rates of vaginal deliveries than white or black mothers born in the US (43). Despite risk factors such as advanced maternal age, late prenatal care initiation, poor nutrition, and anemia, the incidence of low birthweight infants and prematurity were not higher among Hmong refugee women than among US-born mothers (41). A similar pattern was observed in a more recent study of refugees from various ethnic groups. As previously mentioned, when compared to US mothers, refugee women had a lower risk of having preterm infants (ARR = 0.67, 95% CI = 0.49–0.89, *p* = 0.007) (32). Lower levels of alcohol and tobacco consumption were frequently mentioned as potential contributors to these healthy migrant effects although no empirical study investigated the potential hypothesis (28, 41, 42).

A number of studies documented “unhealthy acculturation” or “unhealthy assimilation” which could be characterized by the adoption of risky health behaviors such as drinking or smoking (57). For example, one study conducted in Sweden, one of the countries with the largest number of refugees per capita in Europe, revealed that smoking during pregnancy increases with duration of residence among migrants (58). Another study also concluded that the level of acculturation is associated with a higher smoking rate among Asian female adults in the US (59). Thus, determining whether the level of smoking or drinking, which are frequently attributed to the health migrant effects among refugee women, increases with length of residence and its influence on maternal health outcomes could be an important topic.

It may also be worthwhile to identify various factors that may contribute to generally favorable maternal health outcomes, such as dietary practices and health promotion practices among refugee women. Much is known about factors contributing to Mexican women's healthy migrant effects. Traditional dietary practices, lower levels of tobacco and alcohol consumption, stronger family support, and stronger religious beliefs and practices, for example, were found to be protective factors for favorable perinatal outcomes for Mexican women in one study (60). However, other than lower levels of smoking and drinking, potential protective factors for refugee women are largely unknown.

### 5.5. Resistance to obstetric interventions and its association with maternal health outcomes

Resistance to obstetric interventions such as C-sections and induced delivery, as well as refugee women's mistrust of the health care system and health care providers, has been well documented, particularly among Somali refugee women. Nevertheless, other from a few anecdotal cases reported by health care providers, there is little data on how the resistance affects maternal health outcomes (39).

It is inconclusive whether the prevalence of cesarean delivery is higher among refugee women than among US-born mothers although it is perceived as unacceptably high among Somali communities (37). Thus, more research may be required to determine the current status of obstetric interventions in comparison to mothers in the host country, as well as whether interventions for a specific group are higher than for any other group, and whether resistance to obstetric interventions is associated with adverse maternal health outcomes. As Somali women and men believe that obstetric interventions are the result of a lack of knowledge or training for women with FGC (37), lucrative motives, or rushing practices, more research on the current status, practices, and potential implications in collaboration with communities could help alleviate some of the misunderstanding and distrust.

### 5.6. Unspoken needs and concerns

According to one study in the scoping review, “gracious acceptance” or “unquestioning agreeability” among refugee women may not always translate into their satisfaction with health care, and there may be some unspoken needs and concerns due to refugee women's reluctance to voice a negative opinion and social hierarchy embedded in the doctor-patient relationship in a particular culture (53). They may be hesitant to ask questions due to lack of knowledge of their rights, and fear potentially associated with their traumatic experiences under dictatorship (53). To solicit their unspoken concerns and questions, it might be useful to get perspectives from others such as doulas or family members who may be able to reveal “insider knowledge” while equipping and empowering refugee women with knowledge and skills (53). For rich discussions with those with limited language proficiency and educational levels, different elicitation strategies, such as video elicitation prior to a focus group discussion, could also be used (37).

### 5.7. Maternal country of birth as a proxy for refugee status

In some studies, especially when using secondary data, maternal country of birth was frequently used as a proxy for refugee status (32, 43). In one study, women from Burundi, the Democratic Republic of the Congo, Eritrea, Rwanda, and Somalia were considered refugees (43). Despite the large number of refugees from those countries, not all migrants are refugees, and they include people of various socioeconomic backgrounds, language proficiency, perspectives, and values. The ways in which they navigate and experience the healthcare system may also differ, potentially leading to different health outcomes (9).

### 5.8. Neglected ethnic groups

Certain refugees are more visible than others. Some minorities may be overlooked despite pressing needs. For example, the US Census and many other demographic questions in many major studies classify people from the Middle East and North Africa, including refugee women from Iran, Iraq, Syria, or Afghanistan, as White individuals. However, the majority of people from these regions consider themselves to be an ethnic minority in the US (61). Invisibility and underrepresentation in studies and figures in reports frequently indicate ignorance about their status and needs, as well as a lack of funding or programs dedicated to the population. To compare the levels of need across ethnic groups, it may be necessary to include many, if not all, ethnic groups in studies. Furthermore, in large studies conducted at the state or national level, migration status, including “refugeeness,” could be included, or refugee women could be purposefully sampled in different assessments conducted to assess the health and wellbeing of women, mothers, caregivers, and children.

### 5.9. Macro-level factors not only micro-level factors

Although macro-level factors such as state-level policies and community-level resources influence maternal health care access and utilization and maternal health outcomes, a limited number of studies shed light on the levels and all the others only focused on the individual level factors. To fully comprehend the health disparities among immigrants, meso- and macro-level factors that cause and reproduce health disparities among immigrants must be better understood (62, 63). A variety of macro-level factors such as immigration policy, perceived discrimination, built environment, neighborhood ethnic composition, the discrepancy between origin and host environment cultures, housing, food supply, transportation, weather, and policy and available resources can be substantially influential for the health of refugees and should be part of the inquiry when studying the health of refugees.

### 5.10. Limitations

One limitation of this scoping review is that we did not include studies on general immigrants from countries that send many refugees to the US, such as Somalia, nor did we actively seek studies on immigrants from those countries or ethnic groups, such as the Hmong, for some reason. First, people from those countries may have different immigration statuses, and their experiences may differ from those of refugees. According to one observational study, migrant women with refugee backgrounds from African regions are at a higher risk of adverse pregnancy outcomes than migrant women without a refugee background although they are from the same regions (64). Furthermore, immigrant women are more likely to stay longer, and acculturation is strongly related to length of residence in a country and health outcomes (60). As a result, we concentrated our efforts on refugees rather than broadening our search to include other immigrant groups. Another limitation is that this scoping review did not include gray literature. Although there are some discussions in the form of gray literature published by refugee resettlement agencies or other organizations that serve the population, the goal of this scoping review was to delineate the scope and magnitude of the academic evidence bases and identify the evidence gap on maternal health among refugee women resettled in the US so that further research could be better informed. Finally, because we included all published studies, including studies from several decades ago, some characteristics described in studies from the 1980's or 1990's may not reflect current norms.

## 6. Conclusions

The scoping review emphasizes the critical need for early prenatal care initiation and more frequent prenatal care visits among refugee women. Sensitization and raising awareness to change their perceptions, as well as interventions such as reminder calls or transportation assistance, could help promote prenatal care visits and early initiation of prenatal care among refugee women in the US. Furthermore, more needs to be done to dispel some of the myths and reduce resistance to obstetric interventions and mistrust. Better communication and engagement of women and their families in decision-making could facilitate this. Despite numerous risk factors, refugee women have generally favorable maternal health outcomes. The mechanism by which healthy migrant effects occur could be better understood, allowing protective factors to be maintained throughout the resettlement and acculturation process. In addition, there were some inconsistencies in terms of utilization of maternal health care services and maternal health outcomes in the literature, which could be partly attributed to the high level of heterogeneity. Given the high level of heterogeneity in refugee populations, disaggregated values should be reported whenever possible. Finally, the scoping review identifies critical gaps in the literature, such as the underrepresentation of different ethnic groups of refugee women in refugee maternal studies in the US. Since this invisibility may indicate unspoken and unaddressed needs, more attention should be paid to underrepresented and understudied groups of refugee women in order to achieve health equity for all.

## Data availability statement

Publicly available datasets were analyzed in this study. This data can be found here: All data generated or analyzed during this study are included in this published article. As the subject of analysis is published articles, and all of the articles are open to public, the articles can be easily searched and retrieved.

## Author contributions

SY conceptualized and designed the study, screened the articles, synthesized the findings, and wrote the manuscript. As a co-reviewer, YP screened the articles and assisted with data analysis. DM provided feedback on the protocol, as well as assistance in developing search strategies and exporting the results. HA provided guidance for designing the protocol and search strategies and overall design. Throughout the process, JE, KE, and PM provided insightful comments and feedback. The final manuscript was read and approved by all authors.
