# Supplementary material for: A scoping review of maternal health among resettled refugee women in the United States
Source: Front Public Health. 2023 May 9;11:1157098. doi: 10.3389/fpubh.2023.1157098 (PMC10214470; doi:10.3389/fpubh.2023.1157098)
Supplement: Supplementary file 1 [file Table_1.docx]

**Additional File 1. Search Strategies**

**PubMed Search Strategy**

(refugee*[tiab] OR resettled) AND (matern* OR "maternal health" OR "maternal care" OR prenatal OR prenatal OR antenatal OR perinatal OR intrapartum OR postpartum OR post-partum OR postnatal OR childbirth OR obstet* OR pregnan* OR reproductive OR puerperium OR antepartum)

**CINAHL Search Strategy**

S1 TI refugee* OR AB refugee*

S2 resettled

S3

matern* or maternal care or maternal health or prenatal or prenatal care or prenatal care or perinatal care or antenatal or perinatal or intrapartum or postpartum or post partum or postnatal or childbirth or obstet* or pregnan* or reproductive or puerperium or antepartum

S4

(matern* or maternal care or maternal health or prenatal or prenatal care or prenatal care or perinatal care or antenatal or perinatal or intrapartum or postpartum or post partum or postnatal or childbirth or obstet* or pregnan* or reproductive or puerperium or antepartum) AND (S1 OR S2)

**PsycINFO Search Strategy**

S1

TI refugee* OR AB refugee*

S2 resettled

S3

matern* or maternal care or maternal health or prenatal or prenatal care or prenatal care or perinatal care or antenatal or perinatal or intrapartum or postpartum or post partum or postnatal or childbirth or obstet* or pregnan* or reproductive or puerperium or antepartum

S4

(matern* or maternal care or maternal health or prenatal or prenatal care or prenatal care or perinatal care or antenatal or perinatal or intrapartum or postpartum or post partum or postnatal or childbirth or obstet* or pregnan* or reproductive or puerperium or antepartum) AND (S1 OR S2)

**EMBASE Search Strategy**

1 'refugee*':ab,ti

2 resettled

3 matern* OR 'maternal care' OR prenatal OR prenatal OR 'prenatal care' OR antenatal OR perinatal OR intrapartum OR postpartum OR 'post partum' OR postnatal OR childbirth OR obstet* OR pregnan* OR reproductive OR puerperium OR antepartum

4 1 or 2

5 3 and 4
